# The International Health Regulations (2005), the threat of populism and the COVID-19 pandemic

**DOI:** 10.1186/s12992-020-00600-4

**Published:** 2020-07-28

**Authors:** Kumanan Wilson, Sam Halabi, Lawrence O. Gostin

**Affiliations:** 1 Department of Medicine, University of Ottawa, Bruyere Research Institute and Ottawa Hospital Research Institute, Civic Campus, 1053 Carling Avenue, Box 684, Administrative Services Building, Ottawa, ON K1Y 4E9 Canada; 2 Center for Global Health Science and Security, Georgetown University Medical Center, NW 306, Medical-Dental Building, 3900 Reservoir Road, N.W., Washington DC, 20007 USA; 3 Georgetown Law, Georgetown University, 600 New Jersey Avenue NW, Washington DC, 20001 USA

**Keywords:** Health policy, International health, Populism

## Abstract

The global response to the COVID-19 pandemic has laid bare weaknesses and major challenges in the international approach to managing public health emergencies. Populist sentiment is spreading globally as democratic nations are increasing their support for or electing governments that are perceived to represent “traditional” native interests. Measures need to be taken to proactively address populist sentiment when reviewing the IHR (2005) effectiveness in the COVID-19 pandemic. We discuss how populism can impact the IHR (2005) and conversely how the IHR (2005) may be able to address populist concerns if the global community commits to helping states address public health threats that emerge within their borders.

## Background

The global response to the COVID-19 pandemic has laid bare weaknesses and major challenges in the international approach to managing public health emergencies. Specifically, the International Health Regulations (IHR (2005)), the primary law governing the global response to such events, did not appear to succeed in its objectives – controlling the spread of a severe public health threat while avoiding unnecessary interference with international traffic and trade. As the pandemic spreads and national economies decelerate, scholars, public health officials, and world leaders are asking whether the situation may have been different if countries had fulfilled their legal obligations to both report to the World Health Organization more robustly and follow its travel and trade guidance more faithfully. These questions are all the more urgent given the populist resistance that has mounted against public health measures in some countries, including rapid re-opening of the economy and general lack of acknowledgement of the extent of the threat within their borders [1].

While several reasons may explain the apparent failure of the IHR (2005) attention should be focused on the circumstances that are likely to envelop its review and revision as a sixth and by far most severe public health emergency of international concern – the legal triggering language of the IHR (2005) – afflicts the world. The IHR (2005) were formulated during an epoch of optimism in global institutional cooperation. In contrast, at present populist sentiment has been spreading globally over the past decade as nations are increasing their support for or electing governments that are perceived to represent “traditional” native interests [2]. As the world emerges from the COVID-19 pandemic efforts to reconstruct a new global public health world order will need to account for the populist skepticism that now faces evidence-based public health measures, a skepticism that has resulted in delayed responses to the pandemic, underplaying of the risk and thus potentially contributed to avoidable illness and death [1].

In this commentary we discuss the potential impact of populist/nativist sentiment on the IHR (2005), how this was manifested during the response to the COVID-19 pandemic and how populist concerns should be addressed when re-evaluating the IHR (2005) in the aftermath of the pandemic.

## Main text

### Populist sentiment and global governance

Populism refers to movements which appeal to local populations who believe that their needs are not prioritized by ruling elites [3]. An important aspect and contributor to rising populist sentiment is engagement of nations in international agreements (e.g., the Paris Climate Agreement, multilateral trade agreements), intergovernmental institutions (e.g., United Nations and specialized agencies like the World Health Organization), and transnational alliances (e.g., European Union, NATO). Fueling the disenchantment is the belief that these regimes subordinate the interests of nation states to those of the international community and that the national populace does not have a voice in the decision-making process but rather that these decisions are being made by unaccountable elites [4].

The Brexit controversy and the United States’ withdrawal from its commitments to the Paris Climate Agreement are two tangible impacts of the rising populism reacting to international governance. Somewhat less discussed is the potential impact of populism on highly impactful health arrangements such as the WHO’s International Health Regulations (2005) [5, 6]. The IHR (2005) impose significant requirements upon States Parties and some suggest the treaty represents one of the most invasive international agreements vis-à-vis impact on national sovereignty [7]. Thus, they have the potential to trigger many of the populist concerns expressed over other international governance arrangements. With the response to COVID-19 some of these concerns have become more evident as local protests against social distancing measures proliferate as does skepticism of foreign countries and international institutions.

### IHR (2005) national sovereignty and national interests

The IHR (2005) include requirements for the development of States Parties’ capacity to rapidly identify, report, and respond to potential public health emergencies of international concern (PHEIC). As the IHR were approved unanimously by the World Health Assembly, these requirements are legally binding on countries with the exception of some technical exceptions called “reservations.” The IHR also provides WHO with the authority to independently collect surveillance data on potential PHEICs within a country’s borders, report this information to other potentially affected countries, and to issue recommendations, such as trade and travel advisories, to control the spread of these threats [8].

Like most international agreements, there are no formal penalties associated with non-compliance. Some low- and middle-income countries argue that they lack the resources necessary to fulfill the agreement’s mandates and some large, middle income countries have deviated from WHO recommended measures precisely because of the economic effect of those measures. Indeed, some suggest this agreement is particularly advantageous for wealthier countries, who are better able to mobilize their own resources and do so more quickly should a global health threat be reported through IHR mechanisms. For poorer countries, the IHR requirements potentially divert limited public health financial and human resources from tackling domestic public health threats such as HIV, tuberculosis and malaria which have a more immediate impact on the health of their populations. Furthermore, reporting public health events has in past epidemics led to the imposition of unjustified trade and travel restrictions upon reporting countries [9, 10].

Compliance with the IHR could thus stoke two kinds of populist resentment. First, reporting notifiable events, while potentially negatively affecting any country resulting in reticence to report, may disproportionately burden poorer constituencies [11]. For example, reporting of a possible animal borne illness that has the potential to cross international borders could be viewed as having a negative impact on farmers and local markets in poor countries for the (speculative) benefit of consumers in richer countries. This has emerged as a concern with the reluctance of China to close its wet markets, a potential source of the current pandemic [12]. Given that in the early stages of evaluating potential threats there is often uncertainty as to the nature and extent of the threat, decisions to report involve some level of discretion. While the National Focal Point (NFP) of a State Party is expected to report such events, other ministries may be required under national law to approve such a notification. Thus, conflicts may occur where a ministry perceives that an event does not meet reportable criteria but the NFP perceives that it does. In these situations, there is a risk that reporting an event could stoke populist sentiment – particularly the view that a national government is prioritizing its international commitments over the interests of its local population. This burden may weigh disproportionately on less well-off citizens within countries.

Second, the IHR necessarily imposes obligations on national governments, which ostensibly could pose trade-offs with authority generally enjoyed by subnational, provincial or state governments. Many of the IHR requirements related to detection, reporting and response may constitutionally lie with local or state/provincial governments and may not fall under national competencies. In systems where local or state/provincial governments are viewed as more responsive or legitimate to local needs and interests, the conflict may generate populist resistance to international health agreements, especially where local and national authorities disagree about reporting an event [13, 14].

### Populism and the response to COVID-19

Distrust of the WHO and global approaches to managing pandemics amongst some nations have been highlighted in the response to COVID-19 [1]. Issues related to the transparency of States Parties and their willingness to report have emerged, particularly with respect to disclosure surrounding the original outbreak in Wuhan, China. The WHO has also been accused of delaying the declaration of a PHEIC and not calling for needed travel restrictions [15, 16]. Virtually every country in the world has exceeded WHO guidance on travel. Partly as a consequence of populist sentiment combined with distrust of the WHO, the United States has announced its decision to cease funding of the organization [17]. Further evidence of the impact of populist sentiment is the reluctance of countries with purportedly populist governments, such as the United States, Brazil and the United Kingdom, to follow WHO guidance. In the UK this was manifest by the initial “herd immunity’ as opposed to “lockdown” strategy. In the United States and Brazil there have been efforts to downplay the impact of the pandemic and question international guidance. In the United States the rapid re-opening and subsequent resurgence in cases is illustrative of the negative consequences of not following international guidance [1]. At the same time it needs to be acknowledged that the WHO guidance on both not imposing travel restrictions and questioning the value of masks at the outset of the pandemic may not have been supported by subsequent evidence.

### Managing the populist threat to the IHR

There are characteristics of the IHR that distinguish it from international regimes that have faced more significant populist backlash. First, the benefits of compliance are more immediate and tangible than with, for example, the Paris Climate Agreement or international economic arrangements. In exchange for participation, the World Health Organization and member states can and do offer assistance to prepare for and manage emerging public health threats—even though in practice international assistance to lower-income countries to build core IHR capacities has lagged [18]. This assistance can include training and capacity development as well as support or surveillance, reporting and response initiatives, Second, serious public health threats by their nature adversely affect local populations and economies, so having an international coordinating mechanism to help when those threats materialize resonates with a kind of intuitive understanding at all levels – local, national, and global.

There are several steps that can be taken to proactively address populist sentiment as revision of the IHR (2005) potentially nears. First, the decision to declare a PHEIC must be made after a transparent and accountable process; so must decisions to recommend travel and trade measures. Past declarations have been vulnerable to criticisms that decisions were made behind closed doors, by unaccountable bureaucrats, with potential conflicts of interest inadequately addressed [19]. This emerged again, as a point of criticism with the declaration of the COVID-19 public health emergency.

Second, WHO and the broader international community must rapidly provide materially more support to help States Parties to address reported PHEICs to ensure that the local populace has a visible understanding of the reciprocal nature and benefits of the IHR. Consideration should be given to the creation of a compensation fund for States Parties that are adversely economically affected by timely reporting of potential threats.

Third, the local populations that are potentially most directly impacted by IHR decision-making must have awareness of how their country is managing and reporting on local threats and have an opportunity to express their concerns about how the public health threat and any actions to control its international spread may impact them. It is this perception that the “so-called elites” are not interested in these local issues that perhaps, more than any other factor, has fueled the resentment of participating in international pacts [4].

## Conclusion

Highly transparent and positive local, national and global responses to international health threats through the IHR can help assure global health security and do so in ways that are visible and valued by local populations. As the world re-evaluates the IHR in light of the COVID-19 pandemic, revisions to how global health threats are managed, instead of stoking populist concerns, could alleviate these concerns by demonstrating the tangible benefits of participating in the global community.

## Data Availability

Not applicable.
